# Association of nutritional indices with hyporesponsiveness to hypoxia-inducible factor-prolyl hydroxylase inhibitors in patients with chronic kidney disease: a single-center retrospective observational study

**DOI:** 10.1186/s12882-026-05074-x

**Published:** 2026-06-16

**Authors:** Takuya Minamishima, Hiroyuki Mizoguchi, Nobuhiro Nishibori, Hiroaki Ikesue

**Affiliations:** 1 Department of Hospital Pharmacy, Nagoya University Hospital, Nagoya, Japan; 2 Department of Nephrology, Nagoya University Graduate School of Medicine, Nagoya, Japan

**Keywords:** Chronic kidney disease, Renal anemia, Hypoxia-inducible factor-prolyl hydroxylase inhibitor, Nutritional indices, Controlling nutritional status score

## Abstract

**Background:**

Hypoxia-inducible factor-prolyl hydroxylase inhibitors (HIF-PHIs) are promising therapeutic alternatives for renal anemia because their hematopoietic effects are less influenced by inflammation. However, HIF-PHIs did not improve anemia in some cases, even at increasing doses. Since non-hemodialysis patients and various HIF-PHIs have not yet been examined, this study aimed to explore the factors associated with HIF-PHI resistance index (HRI) in all patients with chronic kidney disease (CKD) who received HIF-PHIs.

**Methods:**

This single-center, retrospective observational study included patients with dialysis-dependent or non-dialysis-dependent CKD who received HIF-PHIs. Spearman’s rank correlation coefficients were calculated between the HRI and each factor. Factors associated with a high HRI were evaluated using univariate logistic regression analysis, followed by multivariate logistic regression analysis using statistically significant factors as covariates.

**Results:**

The controlling nutritional status (CONUT) score was strongly correlated with HRI (score ≥ 5 [moderate to severe malnutrition] was associated with a high HRI). Further analysis of daprodustat (*n* = 38) showed that this association remained significant after adjusting for potential confounders (OR = 2.2; 95% CI, 1.09–4.44; *p* = 0.028).

**Conclusion:**

Malnutrition, which is closely associated with anemia, may contribute to the reduced responsiveness to HIF-PHIs. A high HRI may be undesirable for any patient because of safety concerns and economic burden.

**Supplementary Information:**

The online version contains supplementary material available at 10.1186/s12882-026-05074-x.

## Introduction

Renal anemia is a common complication of chronic kidney disease (CKD), with prevalence rates of 40% and 60% for stage G4 and G5 CKD, respectively, in the Japanese population [1]. Anemia predicts CKD progression, cardiovascular mortality, and the development of cardiorenal anemia syndrome in patients with CKD [1]. Erythropoiesis-stimulating agents (ESAs) have been used to reduce transfusion requirements. However, issues such as frequent injections, ESA hyporesponsiveness, and potential cardiovascular disease (CVD) risk remain [2]. Hypoxia-inducible factor-prolyl hydroxylase inhibitors (HIF-PHIs), a new class of oral drugs, are noninvasive and enhance endogenous erythropoietin (EPO) production through HIF-α stabilization. HIF-PHIs also lower hepcidin levels and improve iron utilization. Moreover, HIF-PHIs are less affected by inflammation, possibly overcoming the limitations of ESAs. Therefore, they are expected to play a role in ESA-induced hyporesponsive anemia [2, 3].

Compared to ESAs, HIF-PHIs achieve lower peak serum EPO levels, which may reduce CVD and mortality risks [4–6]. Although HIF-PHIs do not increase serum vascular endothelial growth factor (VEGF) levels at clinical doses [7, 8], dose-dependent increases in EPO and VEGF levels have been reported [9]. Additionally, blood and tissue concentrations of HIF-PHIs may differ, and their long-term effects remain unknown. The risk of malignancy may depend on the extent of HIF activation [10]. Other concerns include off-target inhibition of various α-ketoglutarate (AKG)-based metabolic systems [11] and roxadustat-related hypothyroidism [12], although their association with clinical doses is unclear. Consequently, increasing HIF-PHI doses may be undesirable for any patient due to safety concerns and economic burden, making the identification of relevant factors for HIF-PHI responsiveness a pressing issue for clinicians. In this context, the Japanese Society for Dialysis Therapy proposed the HIF-PHI resistance index (HRI) for patients undergoing hemodialysis [13]. Some patients show poor hemoglobin (Hb) response even with increased doses of roxadustat, and lower serum albumin levels tend to correlate with higher HRI, suggesting an association with malnutrition [13].

This study aimed to calculate HRI in patients with dialysis- and non-dialysis-dependent CKD and its relationship with various factors, including nutritional indices.

## Methods

### Study population

Patients with CKD aged ≥ 18 years who were prescribed HIF-PHIs at Nagoya University Hospital between November 1, 2019, and December 31, 2023, were included in the study. The HIF-PHIs included roxadustat, daprodustat, vadadustat, enarodustat, and molidustat. However, vadadustat (*n* = 14), enarodustat (*n* = 3), and molidustat (*n* = 2) were excluded from the analysis due to the limited number of available cases. The exclusion criteria were hematopoietic disorders from hematologic diseases, follow-up < four weeks or unavailable follow-up data, history of chemotherapy, nephrotic syndrome, and liver cirrhosis complicated by hypersplenism.

The study was conducted in accordance with the principles of the Declaration of Helsinki and Ethical Guidelines for Medical and Health Research Involving Human Subjects, and was approved by the Institutional Review Board of Nagoya University Hospital (approval No. 2023 − 0475). As this was a retrospective study based on medical records, neither written nor verbal consent was obtained from participants. Information about the study, including the option to opt out, was publicly disclosed in written form on the Nagoya University Hospital website, and participants or their legal representatives were allowed to decline participation.

### Data collection

All data were retrospectively collected from electronic medical records. The following were examined as exposures and predictors: C-reactive protein (CRP), albumin, blood urea nitrogen to creatinine ratio (BUN/Cr), body mass index (BMI), neutrophil-to-lymphocyte ratio (NLR), platelet-to-lymphocyte ratio (PLR), controlling nutritional status (CONUT) score, prognostic nutritional index (PNI), geriatric nutritional risk index (GNRI), Glasgow prognostic score (GPS), albumin-bilirubin (ALBI) score, transferrin saturation (TSAT), serum ferritin, estimated glomerular filtration rate (eGFR), liver function, abnormal mean corpuscular volume (MCV) (MCV < 80 fL or > 100 fL), and other laboratory values at baseline and the time of evaluation. Baseline was defined as the date of HIF-PHI initiation; if laboratory values were not available on that date, data obtained up to 4 weeks prior were considered acceptable. Laboratory values obtained within 2 weeks before or after the HRI evaluation date were used for all assessments. Additional data included sex, CKD etiology, ESA dose at baseline, age, differential anemia diagnosis, dialysis type, medical history, comorbidities (e.g., hematologic diseases, gastrointestinal bleeding, malignancies, infections), CKD complications, kidney transplantation history, concomitant medications (iron, zinc, carnitine preparations, antacids including proton pump inhibitors, potassium-competitive acid blockers, and histamine H2 receptor antagonists), drug interactions [8], duration of HIF-PHI use, bleeding or CVD events, and adverse effects at the time of evaluation. The ESA resistance index (ERI) was calculated as follows: [weekly ESA dose (IU) / [Hb (g/dL) × body weight (kg)]. Weekly doses of long-acting ESAs were converted to an equivalent dose of erythropoietin alfa: darbepoetin alfa: epoetin beta pegol = 1:200:225 [14, 15]. The ERI was calculated using the mean ESA dose administered during the 8 weeks preceding HIF-PHI initiation.

### Assessment of nutritional status

The CONUT [16], PNI [16], GNRI [16], GPS [17], and ALBI scores [18] were calculated as previously described. Specifically, the CONUT score was calculated by summing the total lymphocyte count (TLC), albumin, and total cholesterol (T-cho) scores as parameters. The PNI was calculated using albumin and TLC; the GNRI using albumin, body weight, and ideal body weight; the GPS using CRP and albumin; and the ALBI score using total bilirubin and albumin.

### Outcome measures

HRI was calculated as previously described: [weekly HIF-PHI (µg)] / [Hb (g/dL) × body weight (kg)] [13, 19, 20]. For the sensitivity analysis, the dose and dose-to-Hb ratio (dose/Hb) were calculated. High HRI was considered indicative of hyporesponsiveness. HRI > 70 (dose: 6 mg/day, body weight: 60 kg, Hb: 10.0 g/dL) and HRI > 500 (dose: 100 mg/day, body weight: 60 kg, Hb: 10.0 g/dL) indicate hyporesponsivess to daprodustat and roxadustat, respectively. These doses were one step above the lowest recommended starting dose indicated by the Japanese package insert. Body weight was based on the median of this study population (60.1 kg), and Hb levels were based on the lower limit of the target range (10.0 g/dL) according to the Evidence-based Clinical Practice Guideline for CKD 2023 [21]. Body weight measured after hemodialysis was used for such patients. Each attending physician determined the target Hb level for HIF-PHI dose adjustment.

The HRI evaluation date was defined as a single time point during the maximum HIF-PHI dose period, at least four weeks after initiation or dose adjustment, when blood tests, including anemia evaluation, were performed, no bleeding events or transfusions had occurred within one month, and the date exceeded the prior ESA dosing interval. In order to ensure accurate assessment, patients with acute infections or clinical conditions requiring surgical intervention were evaluated only when their condition was stable. The concomitant use of ESAs was not allowed during the administration of HIF-PHIs. To exclude iron deficiency, periods when TSAT was < 20%, ferritin was < 100 ng/mL, and Hb was < 10.0 g/dL, without iron repletion, were not considered [22]. When the Hb level increased by ≥ 2.0 g/dL at four weeks after initiation or dose adjustment, the doses were also excluded based on the dose reduction or interruption criteria described in the Japanese package inserts, and a different period, such as before the dose increase, was used instead. This description in the package inserts is intended to avoid thromboembolic events that may be triggered by a rapid increase in blood viscosity. When multiple evaluation dates were available, the date with the highest HRI was selected.

### Statistical analysis

Patient characteristics are summarized as medians (IQRs) or n (%). The normality of continuous variables was assessed using the Shapiro–Wilk test. Spearman’s rank correlation coefficient (*r*_*s*_) was used in order to assess the correlations between the HRI and each factor at evaluation, as well as between the CONUT score and body weight. Univariate logistic regression was performed with high HRI values as the dependent variable to estimate the OR and 95% CI. Additionally, we performed a median-split analysis of the HRI and linear regression using ln(HRI) as the outcome variable. Statistically significant factors were included as covariates in multivariate analysis using a forced entry method to identify potential confounders. The events per variable (EPV) in the multivariate analysis were set to ≥ five [23]. Multicollinearity among explanatory variables was assessed using the variance inflation factor (VIF). A complete case analysis was used to handle missing data. In the roxadustat group, TSAT was missing in five cases, and ferritin was missing in one case at the evaluation point; therefore, they were excluded. There were no cases where both TSAT and ferritin levels were missing in the absence of iron repletion. Receiver operating characteristic (ROC) curve analysis was performed in order to determine the cut-off value of the CONUT score for high or above-median HRI. This was determined as the point that maximized the sum of sensitivity and specificity. The C-statistic was calculated using the area under the curve (AUC).

A two-sided *p* value < 0.05 was considered statistically significant for all analyses. Statistical analyses were performed using EZR version 1.63 (graphical user interface for R; The R Foundation for Statistical Computing, Vienna, Austria) [24] and IBM SPSS Statistics (version 29.0; SPSS Japan, Inc., Tokyo, Japan).

## Results

### Baseline characteristics

**Fig. 1:**
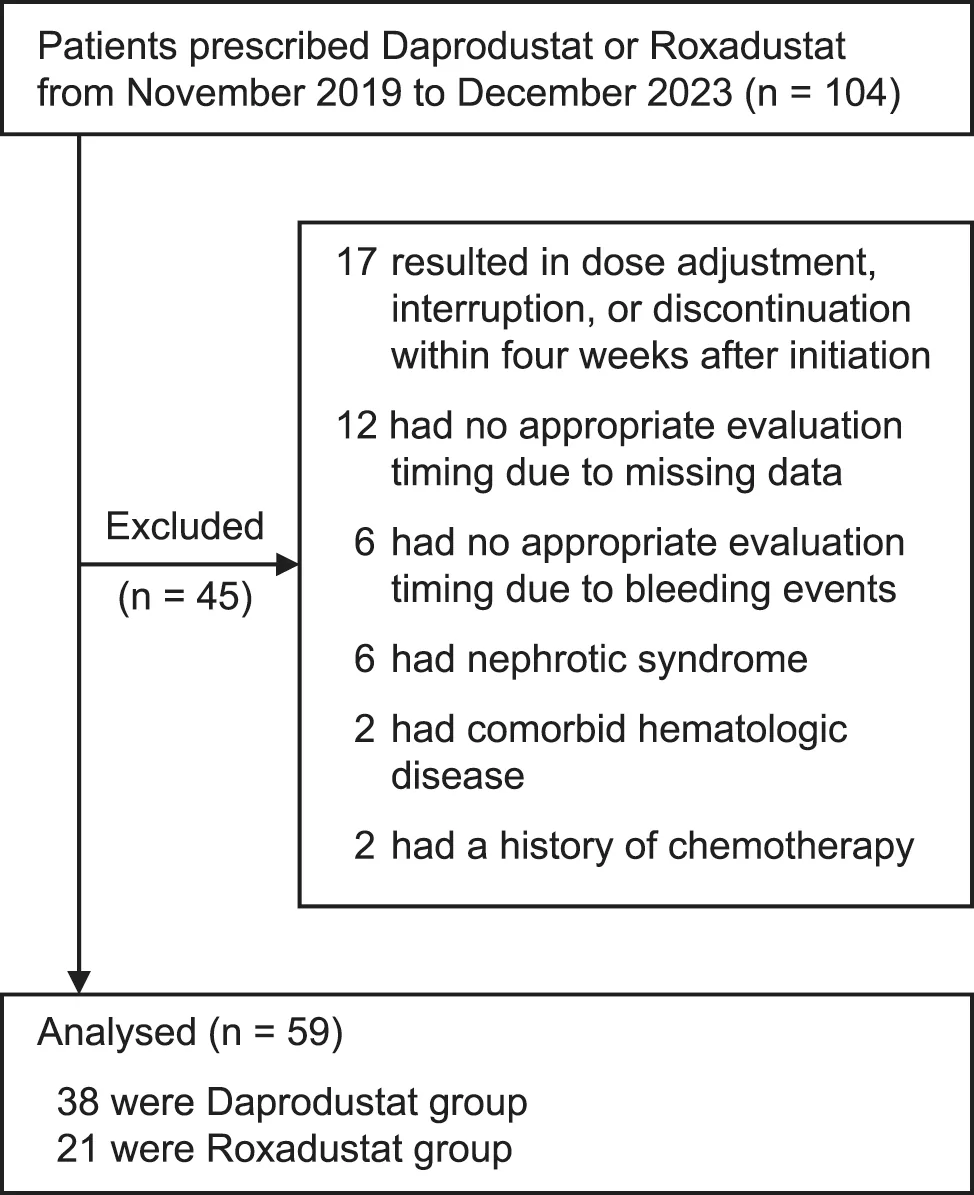
Flow chart of patient selection

Of the 104 patients prescribed HIF-PHIs, 59 were included in the analysis (Fig. 1). We excluded 17 patients with treatment changes within four weeks of HIF-PHI initiation due to dose adjustment, interruption, or death; 12 for inappropriate evaluation timing due to missing data; 6 due to bleeding; 6 with nephrotic syndrome; 2 with comorbid hematologic disease; and 2 with chemotherapy history. Table 1 shows the characteristics of patients in the daprodustat (*n* = 38) and roxadustat (*n* = 21) groups.

**Table 1:** Baseline characteristics of daprodustat and roxadustat groups

	Daprodustat (*n* = 38)	Roxadustat (*n* = 21)
Age [years]	68 (57–82)	66 (59–80)
≥ 75 years [n]	16 (42.1)	7 (33.3)
Female [n]	14 (36.8)	5 (23.8)
Baseline eGFR [mL/min/1.73 m^2^]	12.2 (7.6–17.8)	8.4 (4.1–14.8)
< 15 mL/min/1.73 m^2^ [n]	25 (65.8)	16 (76.2)
≥ 15– < 30 mL/min/1.73 m^2^ [n]	11 (28.9)	1 (4.8)
≥ 30 mL/min/1.73 m^2^ [n]	2 (5.3)	4 (19.0)
Baseline Hb [g/dL]	9.9 (9.3–10.4)	9.5 (8.7–10.3)
Baseline Ferritin [ng/mL]	134 (101–226)	197 (117–306)
Baseline TSAT [%]	27.3 (23.7–35.8)	23.3 (15.0–33.5)
Baseline BMI [kg/m^2^]	24.5 (19.8–27.7)	21.9 (19.9–25.3)
History of ESA use [n]	20 (52.6)	15 (71.4)
Diabetic comorbidity [n]	17 (44.7)	6 (28.6)
CKD etiology: Diabetes [n]	10 (26.3)	4 (19.0)
CKD etiology: Hypertension [n]	11 (28.9)	5 (23.8)
CKD etiology: AD/GN/Vasculitis [n]	8 (21.1)	6 (28.6)
Peritoneal dialysis [n]	10 (26.3)	9 (42.9)
Hemodialysis [n]	0 (0.0)	3 (14.3)
Kidney transplant [n]	1 (2.6)	0 (0.0)

Data are shown as median (IQR) or n (%). IQR, interquartile range; eGFR, estimated glomerular filtration rate; Hb, hemoglobin; TSAT, transferrin saturation; BMI, body mass index; ESA, erythropoiesis-stimulating agent; CKD, chronic kidney disease; AD, autoimmune disease; GN, glomerulonephritis

### Correlation coefficient between HRI and each factor

**Fig. 2:**
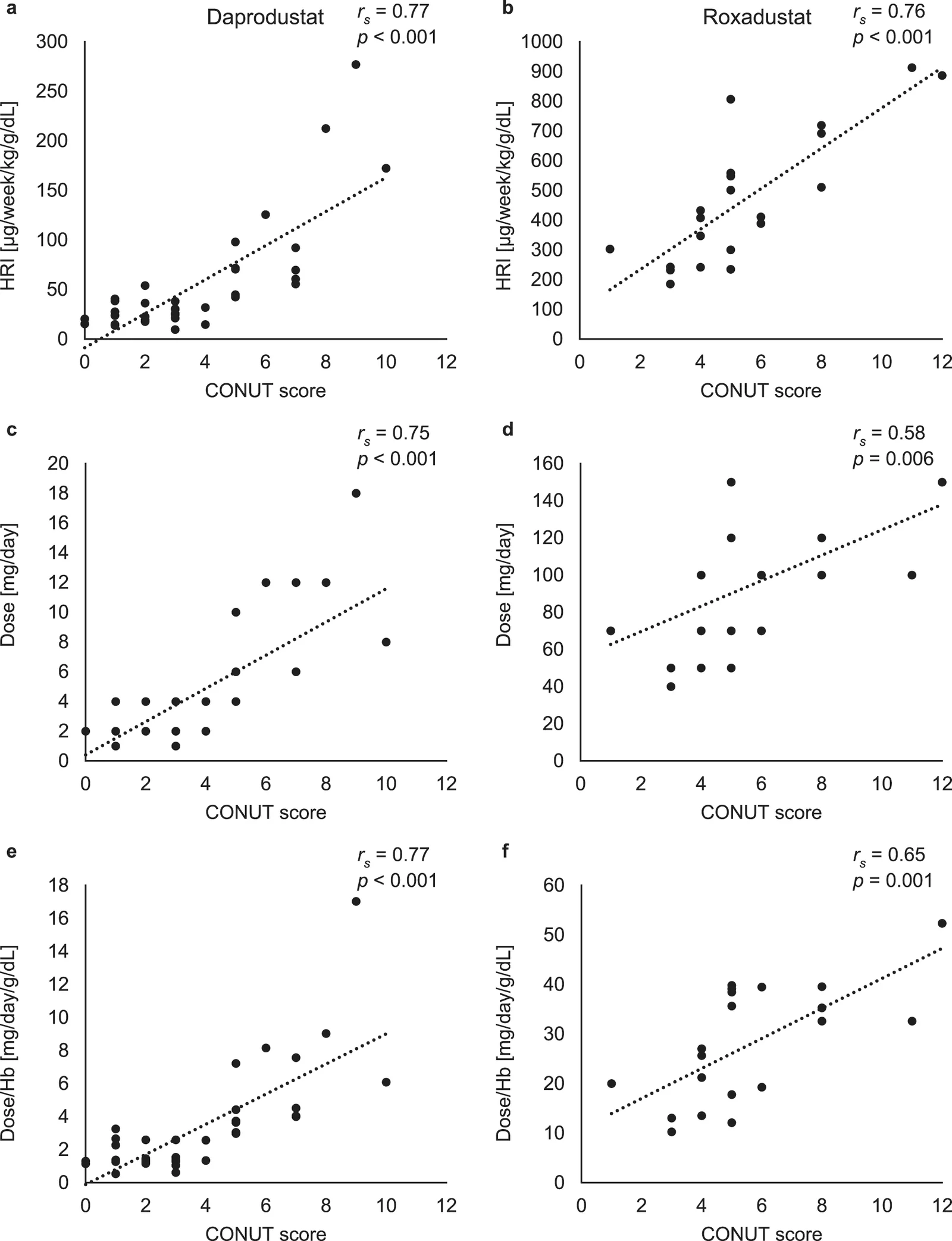
Correlation between CONUT score and HRI of daprodustat (*n* = 38) (**a**), roxadustat (*n* = 21) (**b**), dose of daprodustat (*n* = 38) (**c**), roxadustat (*n* = 21) (**d**) or dose/Hb of daprodustat (*n* = 38) (**e**) and roxadustat (*n* = 21) (**f**). HRI, HIF-PH inhibitor resistance index; CONUT, controlling nutritional status; Hb, hemoglobin; *r*_*s*_, Spearman’s rank correlation coefficient

Several factors were correlated with HRI, and the CONUT score, a nutritional index, showed a strong correlation (*r*_*s*_ > 0.7) (Table 2; Fig. 2a and b). However, no baseline factors showed strong correlations (Supplementary Table S1). Supplementary Table S2 lists the Hb levels, iron-related parameters, and duration of HIF-PHI treatment used to calculate the HRI.

**Table 2:** Correlation coefficient (*r*_*s*_) between HRI and each factor

	Daprodustat		Roxadustat	
	*r*_s_	*p* value	*r*_s_	*p* value
Age	−0.08	0.650	−0.01	0.984
eGFR	−0.46	0.003	−0.05	0.825
BUN/Cr	−0.25	0.124	−0.14	0.531
BMI	−0.14	0.404	−0.03	0.899
NLR	0.35	0.031	0.46	0.040
PLR	0.29	0.079	0.27	0.238
GNRI	−0.48	0.003	−0.04	0.881
CONUT score	0.77	< 0.001	0.76	< 0.001
PNI	−0.67	< 0.001	−0.44	0.047
GPS	0.64	< 0.001	0.34	0.132
ALBI score	0.48	0.002	0.30	0.192
TSAT	0.05	0.762	−0.47	0.062
Ferritin	0.16	0.352	0.12	0.587
BUN	0.50	0.002	0.17	0.461
CRP	0.29	0.077	0.20	0.394
Albumin	−0.52	< 0.001	−0.25	0.275
TLC	−0.60	< 0.001	−0.51	0.018
T-cho	−0.35	0.031	−0.54	0.012
AST	−0.02	0.918	−0.05	0.818
ALT	0.04	0.806	−0.09	0.709
I-Bil	−0.06	0.700	0.03	0.913
T-Bil	0.35	0.033	0.38	0.086
MCV	0.12	0.492	−0.35	0.112

Data are shown as Spearman’s rank correlation coefficient. HRI, HIF-PH inhibitor resistance index; eGFR, estimated glomerular filtration rate; BUN/Cr, blood urea nitrogen to creatinine ratio; BMI, body mass index; NLR, neutrophil to lymphocyte ratio; PLR, platelet to lymphocyte ratio; GNRI, geriatric nutritional risk index; CONUT, controlling nutritional status; PNI, prognostic nutritional index; GPS, Glasgow prognostic score; ALBI, albumin-bilirubin; TSAT, transferrin saturation; CRP, C-reactive protein; TLC, total lymphocyte count; T-cho, total cholesterol; AST, aspartate aminotransferase; ALT, alanine aminotransferase; I-Bil, indirect bilirubin; T-Bil, total bilirubin; MCV, mean corpuscular volume. *r*_*s*_, Spearman’s rank correlation coefficient

### Factors associated with high HRI of daprodustat

In the univariate analysis, CONUT score, baseline eGFR, and ESA usage history were significantly associated. In the multivariate analysis, the CONUT score remained significant (OR = 2.20; 95% CI, 1.09–4.44; *p* = 0.028) (Table 3). These findings are consistent with the sensitivity analyses using the above-median HRI and log-transformed HRI as a continuous variable (Supplementary Tables S3 and S4). Patients undergoing hemodialysis (*n* = 0), kidney transplantation (*n* = 1), or concomitant use of sodium-glucose cotransporter 2 (SGLT2) inhibitors (*n* = 1) were excluded from the analysis.

**Table 3:** Factors associated with the high HRI of daprodustat

	Univariate analysis			Multivariate analysis		
	OR	95% CI	*p* value	OR	95% CI	*p* value
Age ≥ 75 years	0.62	0.13–0.54	0.544			
Female	1.52	0.33–6.96	0.590			
Baseline eGFR [mL/min/1.73 m^2^]	0.85	0.73–0.99	0.041	1.02	0.84–1.25	0.845
Baseline Ferritin [ng/mL]	1.00	0.99–1.01	0.573			
Baseline TSAT [%]	1.09	0.98–1.20	0.088			
Diabetic comorbidity	0.99	0.22–4.43	0.984			
CKD etiology: Diabetes	0.28	0.03–2.56	0.259			
CKD etiology: Hypertension	0.64	0.11–3.68	0.612			
CKD etiology: AD/GN/Vasculitis	2.40	0.44–13.0	0.309			
Peritoneal dialysis	1.57	0.31–7.99	0.586			
Post-cure malignancy	1.37	0.22–8.66	0.737			
History of ESA use	11.3	1.25–103	0.031	6.32	0.4–99.5	0.190
Abnormal MCV	1.79	0.27–11.9	0.548			
Use of antacids	0.88	0.20–3.99	0.871			
Iron therapy	2.46	0.51–11.8	0.260			
Drug interactions	1.37	0.22–8.66	0.737			
CONUT score [per-1 increase]	2.36	1.33–4.21	0.004	2.20	1.09–4.44	0.028

Adjusted odds ratios for the high HRI of daprodustat (*n* = 38) were estimated using logistic regression analysis. Selected covariates were those statistically significant in univariate analysis using the forced entry method. VIF < 5.0, confirming no multicollinearity of the explanatory variables. Abnormal MCV was defined as MCV < 80 fL or MCV > 100 fL. Antacids included proton pump inhibitors, potassium-competitive acid blockers, and histamine H2 receptor antagonists. Drug interactions were due to CYP2C8 inhibition. OR, odds ratio; CI, confidence interval; HRI, HIF-PH inhibitor resistance index; eGFR, estimated glomerular filtration rate; CKD, chronic kidney disease; AD, autoimmune disease; GN, glomerulonephritis; ESA, erythropoiesis-stimulating agent; MCV, mean corpuscular volume; CONUT, controlling nutritional status; VIF, variance inflation factor; CYP, cytochrome P450

### Factors associated with high HRI of roxadustat

In the univariate analysis, only the CONUT score was significantly associated (OR = 3.34; 95% CI, 1.04–10.7; *p* = 0.043) (Table 4). These findings are consistent with the sensitivity analyses using the above-median HRI and log-transformed HRI as a continuous variable (Supplementary Tables S5 and S6). Patients undergoing hemodialysis (*n* = 3), kidney transplantation (*n* = 0), concomitant use of SGLT2 inhibitors (*n* = 1), or drug interactions (*n* = 1) were excluded from the analysis.

**Table 4:** Factors associated with the high HRI of roxadustat

	Univariate analysis		
	OR	95% CI	*p* value
Age ≥ 75 years	0.57	0.08–4.13	0.579
Female	0.86	0.11–6.62	0.882
Baseline eGFR [mL/min/1.73 m^2^]	0.95	0.87–1.04	0.243
Baseline Ferritin [ng/mL]	1.00	1.00–1.00	0.421
Baseline TSAT [%]	1.01	0.94–1.08	0.824
Diabetic comorbidity	4.00	0.54–29.8	0.176
CKD etiology: Diabetes	1.43	0.16–12.7	0.749
CKD etiology: Hypertension	0.86	0.11–6.62	0.882
CKD etiology: AD/GN/Vasculitis	4.00	0.54–29.8	0.176
Peritoneal dialysis	0.50	0.08–2.99	0.448
Post-cure malignancy	6.25	0.84–46.6	0.074
History of ESA use	1.75	0.24–12.6	0.579
Abnormal MCV	0.18	0.02–1.88	0.150
Use of antacids	1.75	0.24–12.6	0.579
Iron therapy	0.36	0.06–2.16	0.262
CONUT score [per-1 increase]	3.34	1.04–10.7	0.043

Odds ratios for the high HRI of roxadustat (*n* = 21) were estimated using logistic regression analysis. Abnormal MCV was defined as MCV < 80 fL or MCV > 100 fL. Antacids included proton pump inhibitors, potassium-competitive acid blockers, and histamine H2 receptor antagonists. OR, odds ratio; CI, confidence interval; HRI, HIF-PH inhibitor resistance index; eGFR, estimated glomerular filtration rate; CKD, chronic kidney disease; AD, autoimmune disease; GN, glomerulonephritis; ESA, erythropoiesis-stimulating agent; MCV, mean corpuscular volume; CONUT, controlling nutritional status

### Cut-off value of the CONUT score

**Fig. 3:**
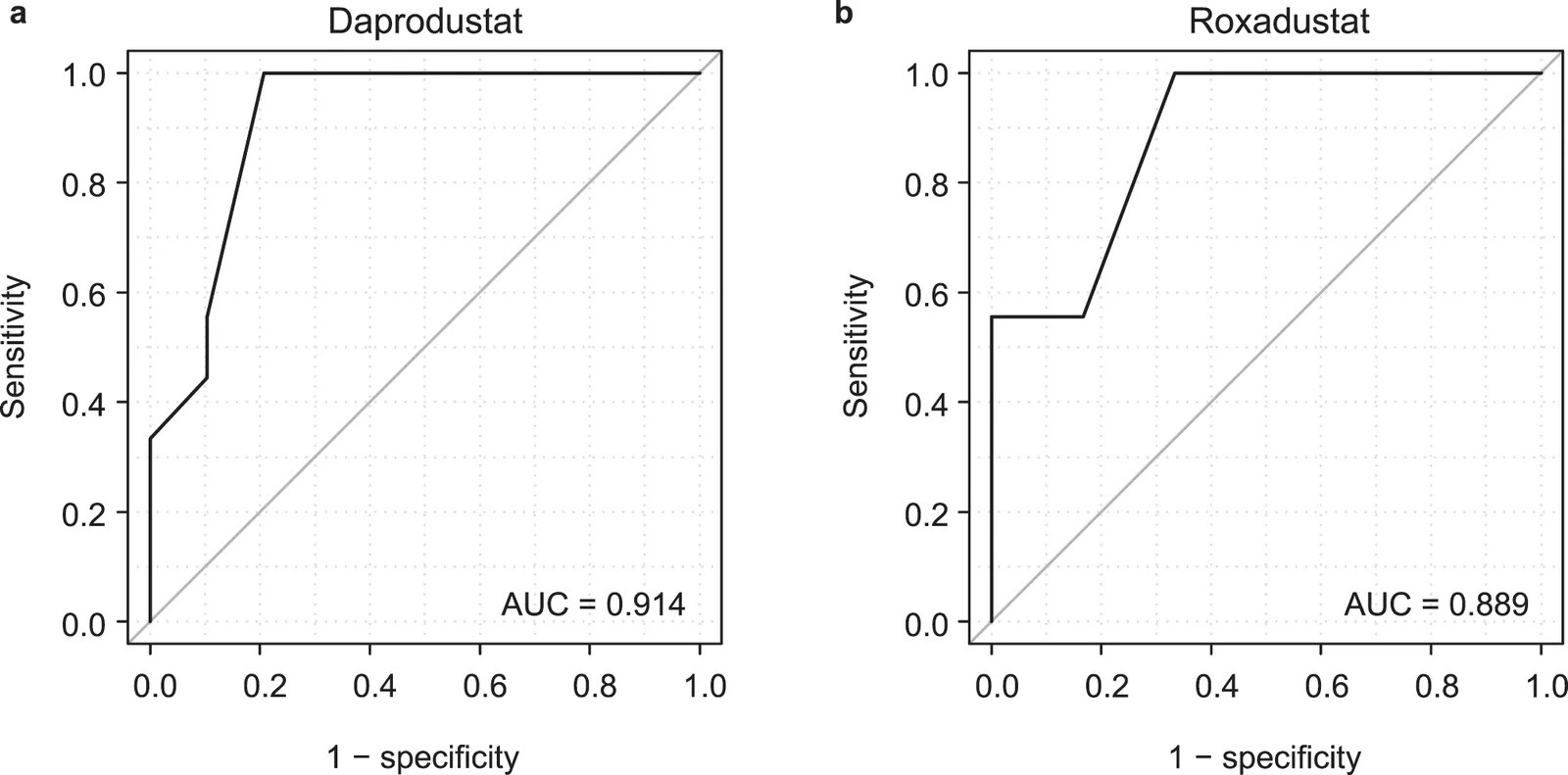
ROC curves of CONUT score for predicting high HRI of daprodustat (*n* = 38) (**a**) and roxadustat (*n* = 21) (**b**). High HRI was defined as HRI > 70 for daprodustat (dose 6 mg/day, body weight 60 kg, Hb level 10.0 g/dL) or HRI > 500 for roxadustat (dose 100 mg/day, body weight 60 kg, Hb level 10.0 g/dL). ROC, receiver operating characteristic; HRI, HIF-PH inhibitor resistance index; CONUT, controlling nutritional status; Hb, hemoglobin; AUC, area under the curve

ROC analysis revealed that a CONUT score of 5, corresponding to moderate malnutrition, yielded the best discriminatory ability between high and low HRI in both groups (Fig. 3). The AUCs exceeded 0.8, indicating a favorable predictive performance. Notably, no cases of high HRI were observed among patients with a CONUT score of < 5 (Fig. 2a and b). In the sensitivity analysis using the above-median HRI, the optimal cut-off value was also a CONUT score of 5, although the AUCs were somewhat lower than those observed in the primary analysis (Supplementary Fig. S1).

## Discussion

To the best of our knowledge, the relationship between HIF-PHI dosage and various nutritional indices has not been previously investigated. The analysis suggested an association between a CONUT score of ≥ 5, indicating moderate or severe malnutrition, and a high HRI, indicating hyporesponsiveness to HIF-PHIs, in patients who received daprodustat or roxadustat. Additionally, for daprodustat, the CONUT score was independently associated with HRI, even after adjusting for confounding factors. Consistent results across the categorical, median-split, and continuous analyses further support the robustness of these findings.

ERI is associated with the risk of all-cause mortality and CVD events [25]. In contrast, no association has been reported between HRI and disease risk or adverse effects. Although HRI has not raised clinical concerns, its potential risks remain unknown. Various factors, including iron deficiency, inflammation, renal function, BMI [26–28], and nutritional deficiencies [29–33], affect ERI. We hypothesized that HRI may also be affected by nutritional status and other related factors. Although the definition of ERI differs between hemodialysis and non-hemodialysis patients [34], we applied a unified definition of HRI because HIF-PHIs are oral drugs with consistent administration routes and intervals.

There are three key differences between our study and those that investigated the factors affecting HIF-PHI dosage. First, we excluded periods of iron deficiency without iron repletion. As iron is essential for erythropoiesis, there is a risk of inappropriate HIF-PHI dose escalation in response to decreased Hb levels when iron deficiency is not corrected [35]. Iron deficiency is associated with thromboembolism; thus, iron supplementation is recommended [22, 28]. The present study population exhibited high adherence to clinical guidelines, and iron deficiency was carefully managed. Similarly, several factors have been reported to affect the responses to HIF-PHIs. For roxadustat, zinc deficiency is considered a potential factor contributing to HIF-PHI dose escalation [36]. A plausible hypothesis is that zinc may be consumed during the course of anemia treatment with HIF-PHIs, and that the patients with low zinc levels may require higher doses. However, in a single-center retrospective study, zinc supplementation did not reduce the HRI [20]. Among other factors, ERI has been suggested to be associated with dose requirements [35, 37].

For vadadustat, in the post hoc analyses of Phase III trials in Japan, independent factors identified as determinants of a better response were as follows: high baseline Hb, low baseline eGFR, high week-20–24 ferritin, and CKD not caused by autoimmune disease/glomerulonephritis/vasculitis in non-dialysis-dependent CKD; and male sex, high baseline CRP, and low baseline ERI in hemodialysis-dependent CKD. These factors have been reported to lead to appropriate dose modifications [19]. However, no such reports have been published for daprodustat alone. Alternatively, one study reported that baseline MCV may be associated with the effects of HIF-PHIs [38]. Second, rather than HRI or related factors at fixed time points, we focused on HRI variability and assessed it when a high HRI was observed after at least four weeks. We examined baseline variables as predictive factors, but none showed strong correlations (Supplementary Table S1). In contrast, our results showed a stronger association with nutritional indices than that previously reported [19]. Third, this is the first report to include daprodustat-focused results. ERI showed a significantly moderate correlation with HRI, dose and dose/Hb in daprodustat (Supplementary Fig. S2), similar to previous findings [19, 35, 37]. Regarding CKD etiology, no association was observed with daprodustat, similar to previous findings on roxadustat dosage (Table 3) [35].

Protein energy wasting, commonly observed in patients with CKD, increases the risk of nutrient deficiencies, including folate, vitamin B12, and iron deficiencies, leading to worsening anemia. Among the various nutritional assessment tools for patients with CKD [16], the GNRI, PNI, and CONUT scores are comprehensive and objective predictors of clinical outcomes in patients undergoing hemodialysis or peritoneal dialysis. Inflammation, liver dysfunction, malignancy, proteinuria, and dialysis can influence albumin levels and thus may not accurately reflect nutritional status [39]. The CONUT score, proposed by the European Society for Clinical Nutrition and Metabolism (ESPEN) in 2003, reflects protein, immune, and lipid metabolism levels. Recent evidence has accumulated for patients with CKD [40–42]. In this study, CONUT scores showed the strongest correlation with HRI (Table 2; Fig. 2a and b), followed by PNI (Supplementary Fig. S3). Although widely recognized, the GNRI may underestimate malnutrition in patients with normal or high BMI due to edema, as it regards low body weight as a malnutrition marker. Unlike the GNRI and PNI, CONUT scores include T-cho, which is inversely associated with ERI [26, 33]. This suggests that reduced lipid levels reflect malnutrition and chronic inflammation, which is considered a reverse epidemiology in patients undergoing hemodialysis [43]. Sensitivity analysis based on previous studies [34] showed that the CONUT score was significantly correlated with the HIF-PHI dose, dose/Hb ratio, and HRI (Fig. 2). In addition, Spearman’s correlation analysis revealed no significant association between the CONUT score and body weight (Supplementary Fig. S4), suggesting that this correlation was not solely due to malnutrition-induced low body weight. CONUT scores are associated with anemia [40, 44], and our findings may reflect not only renal anemia, but also anemia attributable to malnutrition and chronic inflammation. As treating such anemia is difficult, HIF-PHIs, which allow flexible dose adjustments, may frequently be increased in the dosage. Improving the nutritional status and managing chronic inflammation may be associated with lower dose requirements, although future studies should address this causal relationship. Furthermore, HIF-PHIs have been reported to reduce T-cho levels [45], which may result in higher CONUT scores in HIF-PHI users than in non-users. Due to the small number of lipid-lowering drug users, their impact could not be analyzed. However, no significant differences in lipid-lowering drug use across CONUT score categories have been reported [40], suggesting minimal influence. Some studies define CONUT score ≥ 5 as malnutrition [46], which is supported by our findings (Fig. 3 and Supplementary Fig. S1). Given the inconsistent findings regarding nutritional indices for disease outcomes and the absence of specific recommendations in the 2020 Kidney Disease Outcome Quality Initiative (KDOQI) guidelines [47], further research is required to assess nutritional status in patients with CKD. Notably, the CONUT score may reflect inflammatory as well as nutritional status. In our data, the CONUT score showed a statistically significant, albeit modest, correlation with contemporaneous CRP levels (*n* = 59, *r*_*s*_ = 0.40, *p* < 0.01) (data not shown). Although HIF-PHIs are generally considered less susceptible to inflammatory influences, their anti-anemic efficacy may be attenuated [3]. However, in the present study, CRP levels were not significantly correlated with HRI (Table 2).

In the multivariate analysis, CONUT scores were an independent factor associated with high HRI, even after adjusting for ESA usage history and baseline eGFR (Table 3). The higher initial HIF-PHI doses administered to patients with prior ESA, as recommended by the Japanese package insert, may explain the influence of prior ESA use on HRI. Japanese Phase III trials have reported a decline in Hb levels after switching to HIF-PHIs in patients receiving high-dose ESA [48]. Indeed, 35% of patients with prior ESA use had no dose adjustment at the time of evaluation (data not shown). Baseline differences between users and non-users may have also contributed to this finding. Daprodustat and roxadustat are mainly excreted via the hepatobiliary and fecal routes. The association between eGFR and HRI may reflect the preservation of EPO-producing cells that respond to HIF-PHIs in patients with improved residual renal function [49]. However, these findings are inconsistent across studies [19, 35].

This study has several limitations. This was an exploratory, single-center, retrospective analysis limited to the Japanese population. Missing data were excluded, and potential biases, such as institutional prescription patterns, may have influenced the results. In particular, the ROC analysis should be interpreted cautiously, as it is preliminary. Therefore, external validation using independent multicenter datasets is necessary to confirm the predictive performance of the CONUT score for HIF-PHI resistance. The sample size was insufficient for a detailed analysis of other HIF-PHIs or the effects of concomitant medications such as angiotensin-converting enzyme inhibitors/angiotensin receptor blockers [35], statins [50], immunosuppressants [40], and SGLT2 inhibitors, as well as other confounders, including infections. Additionally, given the limited number of patients in the roxadustat group, we considered that performing a multivariate analysis would likely have resulted in overfitting. Therefore, a multivariate analysis for roxadustat was not conducted. Furthermore, nutritional and anemia-related markers, such as folate, copper, zinc, carnitine, vitamin B12, intact PTH, cholinesterase, prealbumin, and hepcidin, or inflammatory markers, such as IL-6, which indirectly cause hepcidin-induced functional iron deficiency, were excluded as these were not measured at the necessary time points. Additionally, nutritional indices that require special techniques were not assessed. To address these limitations and identify reliable predictors, prospective clinical trials should be conducted using analytical rather than descriptive approaches. No predictive factors were identified in this study.

Albumin levels tend to be low in patients with nephrotic syndrome, which is characterized by heavy proteinuria. Nutritional indices that include albumin as a variable, such as the GNRI, are not reliable indices of nutritional status in these patients [39]. Therefore, only a few patients with nephrotic syndrome were excluded from analysis. As no established nutritional assessment method exists in nephrotic syndrome, alternative approaches are necessary.

Regarding safety, D-dimer levels showed no correlation with HRI or the HIF-PHI dose in this study. CVD events occurred in three patients treated with daprodustat (two cerebral infarctions and one subdural hematoma) and in one patient treated with roxadustat (subdural hematoma). Thyroid dysfunction occurred in four patients treated with roxadustat; however, none were associated with HRI (data not shown). No tumor development was observed. Long-term safety beyond one year has not been confirmed in Japan [49], and even in studies with a four-year follow-up, potential risks, such as malignancies, have not been evaluated [45]. As with ESAs [51], it is necessary to investigate whether risks differ depending on whether high HRI is isolated, intermittent, or chronic. Future data should allow for risk evaluation over at least five years [22]. Currently, the Japanese Society of Nephrology [21] and the Asia Pacific Society of Nephrology [22] have issued recommendations for appropriate HIF-PHI use, including cautionary notes regarding potential malignancy risks, thromboembolism, diabetic retinopathy, age-related macular degeneration, and vascular calcification. Higher doses of HIF-PHIs increase drug costs and impose an economic burden.

## Conclusion

This study suggests that CONUT score may be strongly correlated with HRI. The CONUT score is a simple index for nutritional assessment based on laboratory data, and is practical for clinical applications. These preliminary findings may provide useful insights into hyporesponsiveness to HIF-PHIs and contribute to improvements in clinical outcomes, safety, and health economics; however, further studies are warranted to confirm these findings.

## Supplementary Information

Below is the link to the electronic supplementary material.

Supplementary Material 1

## Data Availability

All data generated or analyzed during this study are included in this article and its supplementary material files. Further inquiries can be directed to the corresponding author.

## Abbreviations

HIF: PHI hypoxia-inducible factor-prolyl hydroxylase inhibitor
HRI HIF: PHI resistance index
CKD: Chronic kidney disease
CONUT: Controlling nutritional status
ESA: Erythropoiesis-stimulating agent
CVD: Cardiovascular disease
EPO: Erythropoietin
VEGF: Vascular endothelial growth factor
AKG: α-ketoglutarate
Hb: Hemoglobin
CRP: C-reactive protein
BUN/Cr: Blood urea nitrogen to creatinine ratio
BMI: Body mass index
NLR: Neutrophil-to-lymphocyte ratio
PLR: Platelet-to-lymphocyte ratio
PNI: Prognostic nutritional index
GNRI: Geriatric nutritional risk index
GPS: Glasgow prognostic score
ALBI: Albumin-bilirubin
TSAT: Transferrin saturation
eGFR: Estimated glomerular filtration rate
MCV: Mean corpuscular volume
ERI ESA: Resistance index
TLC: Total lymphocyte count
T-cho: Total cholesterol
r_s_: Spearman’s rank correlation coefficient
EPV: Events per variable
VIF: Variance inflation factor
ROC: Receiver operating characteristic
AUC: Area under the curve
SGLT2: Sodium-glucose cotransporter 2
ESPEN: European society for clinical nutrition and metabolism
KDOQI: Kidney disease outcome quality initiative

## References

[1] Sofue T, Nakagawa N, Kanda E, Nagasu H, Matsushita K, Nangaku M, et al. Prevalence of anemia in patients with chronic kidney disease in Japan: a nationwide, cross-sectional cohort study using data from the Japan Chronic Kidney Disease Database (J-CKD-DB). PLoS ONE. 2020;15:e0236132. 10.1371/journal.pone.0236132. 32687544 10.1371/journal.pone.0236132 PMC7371174

[2] Kuragano T. Treatment of anemia associated with chronic kidney disease: plea for considering physiological erythropoiesis. Int J Mol Sci. 2024;25:7322. 10.3390/ijms25137322. 39000429 10.3390/ijms25137322 PMC11242251

[3] Zhou Y, Chen X-X, Zhang Y-F, Lou J-Z, Yuan H-B. Roxadustat for dialysis patients with erythropoietin hypo-responsiveness: a single-center, prospective investigation. Intern Emerg Med. 2021;16:2193–9. 10.1007/s11739-021-02738-4. 34021853 10.1007/s11739-021-02738-4

[4] Molnar MZ, Tabak AG, Alam A, Czira ME, Rudas A, Ujszaszi A, et al. Serum erythropoietin level and mortality in kidney transplant recipients. Clin J Am Soc Nephrol. 2011;6:2879–86. 10.2215/CJN.05590611. 21980181 10.2215/CJN.05590611 PMC3255371

[5] Czock D, Keller F. Clinical pharmacokinetics and pharmacodynamics of roxadustat. Clin Pharmacokinet. 2022;61:347–62. 10.1007/s40262-021-01095-x. 34905154 10.1007/s40262-021-01095-x PMC8891203

[6] Kuriyama S, Maruyama Y, Honda H. A new insight into the treatment of renal anemia with HIF stabilizer. Ren Replace Ther. 2020;6:63. 10.1186/s41100-020-00311-x.

[7] Akizawa T, Tsubakihara Y, Nangaku M, Endo Y, Nakajima H, Kohno T, et al. Effects of daprodustat, a novel hypoxia-inducible factor prolyl hydroxylase inhibitor on anemia management in Japanese hemodialysis subjects. Am J Nephrol. 2017;45:127–35. 10.1159/000454818. 27978511 10.1159/000454818

[8] Haase VH. Hypoxia-inducible factor-prolyl hydroxylase inhibitors in the treatment of anemia of chronic kidney disease. Kidney Int Suppl (2011). 2021;11:8–25. 10.1016/j.kisu.2020.12.002. 10.1016/j.kisu.2020.12.002 PMC7983025 33777492

[9] Hara K, Takahashi N, Wakamatsu A, Caltabiano S. Pharmacokinetics, pharmacodynamics and safety of single, oral doses of GSK1278863, a novel HIF-prolyl hydroxylase inhibitor, in healthy Japanese and Caucasian subjects. Drug Metab Pharmacokinet. 2015;30:410–8. 10.1016/j.dmpk.2015.08.004. 26643993 10.1016/j.dmpk.2015.08.004

[10] Li J, Haase VH, Hao C-M. Updates on hypoxia-inducible factor prolyl hydroxylase inhibitors in the treatment of renal anemia. Kidney Dis (Basel). 2023;9:1–11. 10.1159/000527835. 36756084 10.1159/000527835 PMC9900466

[11] Nakanishi T, Kuragano T. Growing concerns about using hypoxia-inducible factor prolyl hydroxylase inhibitors for the treatment of renal anemia. Clin Kidney J. 2024;17:sfae051. 10.1093/ckj/sfae051. 38516524 10.1093/ckj/sfae051 PMC10956400

[12] Kouki Y, Okada N, Saga K, Ozaki M, Saisyo A, Kitahara T. Disproportionality analysis on hypothyroidism with roxadustat using the Japanese Adverse Drug Event Database. J Clin Pharmacol. 2023;63:1141–6. 10.1002/jcph.2300. 37408303 10.1002/jcph.2300

[13] Hanafusa N, Abe M, Joki N, Hoshino J, Taniguchi M, Kikuchi K, et al. Ren Replace Ther. 2025;11:54. 10.1186/s41100-025-00646-3. Annual dialysis data report 2022, Japanese Society for Dialysis Therapy (JSDT) Renal Data Registry.

[14] Bonafont X, Bock A, Carter D, Brunkhorst R, Carrera F, Iskedjian M, et al. A meta-analysis of the relative doses of erythropoiesis-stimulating agents in patients undergoing dialysis. NDT Plus. 2009;2:347–53. 10.1093/ndtplus/sfp097. 25949339 10.1093/ndtplus/sfp097 PMC4421401

[15] Vega A, Abad S, Verdalles U, Aragoncillo I, Velazquez K, Quiroga B, et al. Dose equivalence between continuous erythropoietin receptor activator (CERA), darbepoetin and epoetin in patients with advanced chronic kidney disease. Hippokratia. 2014;18:315–8. 26052197 PMC4453804

[16] Yang B, Yang Y, Liu B, Yang M. Role of composite objective nutritional indexes in patients with chronic kidney disease. Front Nutr. 2024;11:1349876. 10.3389/fnut.2024.1349876. 38699544 10.3389/fnut.2024.1349876 PMC11063252

[17] Ștefan G, Zugravu A, Stancu S. Glasgow prognostic score as an outcome predictor for patients initiating hemodialysis. Ther Apher Dial. 2024;28:34–41. 10.1111/1744-9987.14057. 37596836 10.1111/1744-9987.14057

[18] Yang C, Yu Z, Peng B, Mao C, Li J, Cao Y. ALBI grade is associated with clinical outcomes of critically ill patients with AKI: a cohort study with cox regression and propensity score matching. Mediators Inflamm. 2024;2024:1412709. 10.1155/2024/1412709. 39055134 10.1155/2024/1412709 PMC11272401

[19] Nangaku M, Ueta K, Nishimura K, Sasaki K, Hashimoto T. Factors affecting responsiveness of vadadustat in patients with anemia associated with chronic kidney disease: a post-hoc subgroup analysis of Japanese phase 3 randomized studies. Clin Exp Nephrol. 2024;28:391–403. 10.1007/s10157-023-02432-z. 38530490 10.1007/s10157-023-02432-z PMC11033221

[20] Takahashi A. Zinc supplementation enhances the hematopoietic activity of erythropoiesis-stimulating agents but not hypoxia-inducible factor-prolyl hydroxylase inhibitors. Nutrients. 2024;16:520. 10.3390/nu16040520. 38398842 10.3390/nu16040520 PMC10893400

[21] Japanese Society of Nephrology. Essential points from evidence-based clinical practice guideline for chronic kidney disease 2023. Clin Exp Nephrol. 2024;28:473–95. 10.1007/s10157-024-02497-4. 38713253 10.1007/s10157-024-02497-4 PMC11116248

[22] Yap DYH, McMahon LP, Hao C-M, Hu N, Okada H, Suzuki Y, et al. Recommendations by the Asian Pacific society of nephrology (APSN) on the appropriate use of HIF-PH inhibitors. Nephrol (Carlton). 2021;26:105–18. 10.1111/nep.13835. 10.1111/nep.13835 PMC7898910 33222343

[23] Vittinghoff E, McCulloch CE. Relaxing the rule of ten events per variable in logistic and Cox regression. Am J Epidemiol. 2007;165:710–8. 10.1093/aje/kwk052. 17182981 10.1093/aje/kwk052

[24] Kanda Y. Investigation of the freely available easy-to-use software EZR for medical statistics. Bone Marrow Transpl. 2013;48:452–8. 10.1038/bmt.2012.244. 10.1038/bmt.2012.244 PMC3590441 23208313

[25] Panichi V, Rosati A, Bigazzi R, Paoletti S, Mantuano E, Beati S, et al. Anaemia and resistance to erythropoiesis-stimulating agents as prognostic factors in haemodialysis patients: results from the RISCAVID study. Nephrol Dial Transpl. 2011;26:2641–8. 10.1093/ndt/gfq802. 10.1093/ndt/gfq802 21325348

[26] Tanaka S, Kitamura H, Tsuruya K, Kitazono T, Nakano T, FKR Study Collaboration Group. Prevalence, treatment status, and predictors of anemia and erythropoietin hyporesponsiveness in Japanese patients with non-dialysis-dependent chronic kidney disease: a cross-sectional study. Clin Exp Nephrol. 2022;26:867–79. 10.1007/s10157-022-02227-8. 35507237 10.1007/s10157-022-02227-8

[27] Wu HHL, Chinnadurai R. Erythropoietin-stimulating agent hyporesponsiveness in patients living with chronic kidney disease. Kidney Dis (Basel). 2022;8:103–14. 10.1159/000521162. 35527989 10.1159/000521162 PMC9021651

[28] Yamamoto H, Nishi S, Tomo T, Masakane I, Saito K, Nangaku M, et al. 2015 Japanese Society for Dialysis Therapy: guidelines for renal anemia in chronic kidney disease. Ren Replace Ther. 2017;3:36. 10.1186/s41100-017-0114-y.

[29] Rattanasompattikul M, Molnar MZ, Zaritsky JJ, Hatamizadeh P, Jing J, Norris KC, et al. Association of malnutrition-inflammation complex and responsiveness to erythropoiesis-stimulating agents in long-term hemodialysis patients. Nephrol Dial Transpl. 2013;28:1936–45. 10.1093/ndt/gfs368. 10.1093/ndt/gfs368 PMC3707522 23045431

[30] Lee HY, Suh S-W, Hwang JH, Shin J. Responsiveness to an erythropoiesis-stimulating agent is correlated with body composition in patients undergoing chronic hemodialysis. Front Nutr. 2022;9:1044895. 10.3389/fnut.2022.1044895. 36532527 10.3389/fnut.2022.1044895 PMC9755720

[31] Yajima T, Yajima K, Takahashi H. Association of the erythropoiesis-stimulating agent resistance index and the geriatric nutritional risk index with cardiovascular mortality in maintenance hemodialysis patients. PLoS ONE. 2021;16:e0245625. 10.1371/journal.pone.0245625. 33449974 10.1371/journal.pone.0245625 PMC7810304

[32] González-Ortiz A, Correa-Rotter R, Vázquez-Rangel A, Vega-Vega O, Espinosa-Cuevas Á. Relationship between protein-energy wasting in adults with chronic hemodialysis and the response to treatment with erythropoietin. BMC Nephrol. 2019;20:316. 10.1186/s12882-019-1457-0. 31412807 10.1186/s12882-019-1457-0 PMC6694582

[33] Okazaki M, Komatsu M, Shiohira S, Kataoka H, Tsuchiya K, Kawaguchi H, et al. Associations between the erythropoiesis-stimulating agent resistance index and the geriatric nutritional risk index of maintenance hemodialysis patients and increased mortality. Ren Replace Ther. 2015;1:7. 10.1186/s41100-015-0002-2.

[34] Narita I, Hayashi T, Maruyama S, Masaki T, Nangaku M, Nishino T, et al. Hyporesponsiveness to erythropoiesis-stimulating agent in non-dialysis-dependent CKD patients: The BRIGHTEN study. PLoS ONE. 2022;17:e0277921. 10.1371/journal.pone.0277921. 36445882 10.1371/journal.pone.0277921 PMC9707758

[35] Akizawa T, Tanaka-Amino K, Otsuka T, Yamaguchi Y. Factors affecting doses of roxadustat versus darbepoetin alfa for anemia in nondialysis patients. Am J Nephrol. 2021;52:702–13. 10.1159/000519043. 34628408 10.1159/000519043 PMC8686708

[36] Nanmoku K, Ishimitsu A, Abe M, Uchida D, Ando A, Okabe E. Efficacy and factors influencing dosage of roxadustat for erythropoietin-resistant anemia in hemodialysis patients. Nihon Toseki Igakkai Zasshi. 2024;57:197–204. 10.4009/jsdt.57.197.

[37] Akizawa T, Yamaguchi Y, Majikawa Y, Reusch M. Factors affecting the doses of roxadustat vs darbepoetin alfa for anemia treatment in hemodialysis patients. Ther Apher Dial. 2021;25:575–85. 10.1111/1744-9987.13609. 33200512 10.1111/1744-9987.13609 PMC8451884

[38] Odajima K, Arai S, Kido R, Anzai H, Gojo M, Taira S, et al. Erythrocyte indices and response to hypoxia-inducible factor prolyl hydroxylase inhibitors in chronic kidney disease patients with renal anemia: a retrospective study. BMC Nephrol. 2024;25:423. 10.1186/s12882-024-03877-4. 39587465 10.1186/s12882-024-03877-4 PMC11590316

[39] Kang SH, Cho KH, Park JW, Yoon KW, Do JY. Impact of heavy proteinuria on clinical outcomes in patients on incident peritoneal dialysis. BMC Nephrol. 2012;13:171. 10.1186/1471-2369-13-171. 23245677 10.1186/1471-2369-13-171 PMC3538719

[40] Tsuda S, Nakayama M, Tanaka S, Haruyama N, Yoshitomi R, Fukui A, et al. The association of controlling nutritional status score and prognostic nutritional index with cardiovascular diseases: the Fukuoka Kidney Disease Registry Study. J Atheroscler Thromb. 2023;30:390–407. 10.5551/jat.63501. 35811136 10.5551/jat.63501 PMC10067341

[41] Takagi K, Takahashi H, Miura T, Yamagiwa K, Kawase K, Muramatsu-Maekawa Y, et al. Prognostic value of the Controlling Nutritional Status (CONUT) score in patients at dialysis initiation. Nutrients. 2022;14:2317. 10.3390/nu14112317. 35684116 10.3390/nu14112317 PMC9182995

[42] Zhang H, Liu N, Dang H. Association of the Controlling Nutritional Status (CONUT) score with all-cause and cause-specific mortality in patients with diabetic kidney disease: evidence from the NHANES 2009–2018. BMJ Open. 2024;14:e079992. 10.1136/bmjopen-2023-079992. 38653515 10.1136/bmjopen-2023-079992 PMC11043715

[43] Kalantar-Zadeh K, Block G, Humphreys MH, Kopple JD. Reverse epidemiology of cardiovascular risk factors in maintenance dialysis patients. Kidney Int. 2003;63:793–808. 10.1046/j.1523-1755.2003.00803.x. 12631061 10.1046/j.1523-1755.2003.00803.x

[44] Miano N, Di Marco M, Alaimo S, Coppolino G, L’Episcopo G, Leggio S, et al. Controlling Nutritional Status (CONUT) score as a potential prognostic indicator of in-hospital mortality, sepsis and length of stay in an internal medicine department. Nutrients. 2023;15:1554. 10.3390/nu15071554. 37049392 10.3390/nu15071554 PMC10096657

[45] Stoumpos S, Crowe K, Sarafidis P, Barratt J, Bolignano D, Del Vecchio L, et al. Hypoxia-inducible factor prolyl hydroxylase inhibitors for anaemia in chronic kidney disease: a clinical practice document by the European Renal Best Practice board of the European Renal Association. Nephrol Dial Transpl. 2024;39:1710–30. 10.1093/ndt/gfae075. 10.1093/ndt/gfae075 PMC11427073 38573822

[46] Otaki Y, Watanabe T, Shimizu M, Tachibana S, Sato J, Kobayashi Y, et al. Association of malnutrition with renal dysfunction and clinical outcome in patients with heart failure. Sci Rep. 2022;12:16673. 10.1038/s41598-022-20985-z. 36198898 10.1038/s41598-022-20985-z PMC9535020

[47] Ikizler TA, Burrowes JD, Byham-Gray LD, Campbell KL, Carrero J-J, Chan W, et al. KDOQI clinical practice guideline for nutrition in CKD: 2020 update. Am J Kidney Dis. 2020;76:S1–107. 10.1053/j.ajkd.2020.05.006. 32829751 10.1053/j.ajkd.2020.05.006

[48] Akizawa T, Nangaku M, Yonekawa T, Okuda N, Kawamatsu S, Onoue T, et al. Efficacy and safety of daprodustat compared with darbepoetin alfa in Japanese hemodialysis patients with anemia: a randomized, double-blind, phase 3 trial. Clin J Am Soc Nephrol. 2020;15:1155–65. 10.2215/CJN.16011219. 32723804 10.2215/CJN.16011219 PMC7409739

[49] Ogawa C, Tsuchiya K, Maeda K. Hypoxia-inducible factor prolyl hydroxylase inhibitors and iron metabolism. Int J Mol Sci. 2023;24:3037. 10.3390/ijms24033037. 36769359 10.3390/ijms24033037 PMC9917929

[50] Chiang C-K, Yang S-Y, Peng Y-S, Hsu S-P, Pai M-F, Huang J-W, et al. Atorvastatin increases erythropoietin-stimulating agent hyporesponsiveness in maintenance hemodialysis patients: role of anti-inflammation effects. Am J Nephrol. 2009;29:392–7. 10.1159/000169658. 18974640 10.1159/000169658

[51] Atzinger C, Arens H-J, Neri L, Arkossy O, Garbelli M, Jiletcovici A, et al. Hyporesponsiveness to erythropoiesis-stimulating agents in dialysis-dependent patients with anaemia of chronic kidney disease: a retrospective observational study. Adv Ther. 2025;42:471–89. 10.1007/s12325-024-03015-4. 39581908 10.1007/s12325-024-03015-4 PMC11782378

