# Supplementary material for: Association of nutritional indices with hyporesponsiveness to hypoxia-inducible factor-prolyl hydroxylase inhibitors in patients with chronic kidney disease: a single-center retrospective observational study
Source: BMC Nephrol. 2026 Jun 16;27:503. doi: 10.1186/s12882-026-05074-x (PMC13520362; doi:10.1186/s12882-026-05074-x)
Supplement: Supplementary file 1 [file 12882_2026_5074_MOESM1_ESM.docx]

**Supplementary Figure S1.** ROC curves of CONUT score for predicting above-median HRI of daprodustat (n = 38) (a) and roxadustat (n = 21) (b). The median HRI was 37.3 for daprodustat and 411.2 for roxadustat. ROC, receiver operating characteristic; HRI, HIF-PH inhibitor resistance index; CONUT, controlling nutritional status; AUC, area under the curve.

0.0

0.2

0.4

0.6

0.8

1.0

1.0

0.8

0.6

0.4

0.2

0.0

0.0

0.2

0.4

0.6

0.8

1.0

1.0

0.8

0.6

0.4

0.2

0.0

**a**

**b**

Daprodustat

Roxadustat

AUC = 0.878

AUC = 0.841

1 − specificity

Sensitivity

1 − specificity

Sensitivity

**Supplementary Figure S2.** Correlation between ERI and HRI of daprodustat (n = 20) (a), roxadustat (n = 15) (b), dose of daprodustat (n = 20) (c) and roxadustat (n = 15) (d) or dose/Hb of daprodustat (n = 20) (e) and roxadustat (n = 15) (f). HRI, HIF-PH inhibitor resistance index; ERI, erythropoiesis-stimulating agent resistance index; *r_s_*, Spearman's rank correlation coefficient.

**a**

**b**

Daprodustat

Roxadustat

*r_s_* = 0.74

*p* < 0.001

**c**

**d**

*r_s_* = 0.77

*p* < 0.001

**e**

**f**

*r_s_* = 0.70

*p* < 0.001

*r_s_* = 0.38

*p* = 0.17

*r_s_* = 0.22

*p* = 0.424

*r_s_* = 0.23

*p* = 0.404

Dose [mg/day]

| ERI [IU/week/kg/g/dL] | 評価用量 |
| --- | --- |
| 9.70874 | 6 |
| 5.7513 | 2 |
| 12.987 | 6 |
| 1.51347 | 2 |
| 7.60135 | 10 |
| 17.4773 | 18 |
| 3.91822 | 2 |
| 3.58886 | 8 |
| 19.3274 | 12 |
| 9.22282 | 4 |
| 6.29822 | 4 |
| 9.15779 | 4 |
| 6.23891 | 4 |
| 2.45676 | 4 |
| 11.7218 | 12 |
| 12.5828 | 6 |
| 2.58674 | 2 |
| 6.86377 | 2 |
| 14.5631 | 12 |
| 3.80807 | 1 |

Dose/Hb [mg/day/g/dL]

| ERI [IU/week/kg/g/dL] | mHRI |
| --- | --- |
| 9.70874 | 4.51613 |
| 5.7513 | 1.53846 |
| 12.987 | 4.42105 |
| 1.51347 | 1.34615 |
| 7.60135 | 7.21649 |
| 17.4773 | 17.027 |
| 3.91822 | 1.2963 |
| 3.58886 | 6.08696 |
| 19.3274 | 9.03226 |
| 9.22282 | 3.73333 |
| 6.29822 | 3.01075 |
| 9.15779 | 2.66667 |
| 6.23891 | 3.25581 |
| 2.45676 | 2.27642 |
| 11.7218 | 8.15534 |
| 12.5828 | 4 |
| 2.58674 | 1.27273 |
| 6.86377 | 1.37255 |
| 14.5631 | 7.56757 |
| 3.80807 | 0.542636 |

HRI [μg/week/kg/g/dL]

| ERI [IU/week/kg/g/dL] | HRI |
| --- | --- |
| 9.70874 | 69.6933 |
| 5.7513 | 30.5553 |
| 12.987 | 71.0209 |
| 1.51347 | 14.7929 |
| 7.60135 | 98.1836 |
| 17.4773 | 277.088 |
| 3.91822 | 14.9343 |
| 3.58886 | 172.435 |
| 19.3274 | 212.524 |
| 9.22282 | 71.7259 |
| 6.29822 | 44.9031 |
| 9.15779 | 38.5914 |
| 6.23891 | 27.7091 |
| 2.45676 | 40.6504 |
| 11.7218 | 125.66 |
| 12.5828 | 55.7103 |
| 2.58674 | 23.9234 |
| 6.86377 | 24.0798 |
| 14.5631 | 92.2874 |
| 3.80807 | 13.9137 |

HRI [μg/week/kg/g/dL]

| ERI [IU/week/kg/g/dL] | HRI |
| --- | --- |
| 14.2218 | 548.363 |
| 11.0787 | 303.03 |
| 12.4938 | 408.263 |
| 10.7282 | 559.006 |
| 6.73491 | 300.618 |
| 77.7303 | 913.409 |
| 5.7513 | 806.998 |
| 19.4592 | 692.042 |
| 5.88408 | 389.213 |
| 3.64402 | 242.178 |
| 12.5828 | 347.439 |
| 35.4308 | 234.889 |
| 68.0903 | 886.874 |
| 6.27235 | 411.184 |
| 14.5631 | 501.549 |

Dose [mg/day]

| ERI [IU/week/kg/g/dL] | 評価用量 |
| --- | --- |
| 14.2218 | 120 |
| 11.0787 | 70 |
| 12.4938 | 100 |
| 10.7282 | 150 |
| 6.73491 | 70 |
| 77.7303 | 100 |
| 5.7513 | 150 |
| 19.4592 | 100 |
| 5.88408 | 70 |
| 3.64402 | 50 |
| 12.5828 | 100 |
| 35.4308 | 50 |
| 68.0903 | 150 |
| 6.27235 | 100 |
| 14.5631 | 150 |

Dose/Hb [mg/day/g/dL]

| ERI [IU/week/kg/g/dL] | mHRI |
| --- | --- |
| 14.2218 | 35.6436 |
| 11.0787 | 20 |
| 12.4938 | 27.027 |
| 10.7282 | 39.1304 |
| 6.73491 | 17.7966 |
| 77.7303 | 32.6087 |
| 5.7513 | 38.4615 |
| 19.4592 | 35.2941 |
| 5.88408 | 19.2661 |
| 3.64402 | 13.5135 |
| 12.5828 | 25.641 |
| 35.4308 | 12.0968 |
| 68.0903 | 52.3256 |
| 6.27235 | 39.4737 |
| 14.5631 | 39.823 |

**Supplementary Figure S3.** Correlation between PNI and HRI of daprodustat (n = 38) (a), roxadustat (n = 21) (b). HRI, HIF-PH inhibitor resistance index; PNI, prognostic nutritional index; *r_s_*, Spearman's rank correlation coefficient.

*r_s_* = −0.67

*p* < 0.001

*r_s_* = −0.44

*p* = 0.047

**a**

**b**

Daprodustat

Roxadustat

HRI [μg/week/kg/g/dL]

| PNI | HRI |
| --- | --- |
| 33.5 | 69.6933 |
| 45.5 | 30.5553 |
| 39 | 71.0209 |
| 38.5 | 9.66984 |
| 37 | 14.7929 |
| 40.5 | 98.1836 |
| 23.5 | 277.088 |
| 47 | 14.9343 |
| 24.5 | 172.435 |
| 28 | 212.524 |
| 39.5 | 71.7259 |
| 38 | 44.9031 |
| 43.5 | 20.5563 |
| 35.5 | 43.7404 |
| 50 | 22.6978 |
| 45 | 38.5914 |
| 34 | 70.6143 |
| 45 | 27.7091 |
| 39 | 32.03 |
| 43 | 38.267 |
| 43 | 21.3558 |
| 41.5 | 42.5166 |
| 45 | 54.1251 |
| 44.5 | 40.6504 |
| 50 | 15.5073 |
| 36 | 125.66 |
| 48.5 | 24.9288 |
| 33 | 60.7287 |
| 41 | 25.8288 |
| 30.5 | 55.7103 |
| 44 | 36.3825 |
| 49 | 23.9234 |
| 51.5 | 24.0798 |
| 35.5 | 92.2874 |
| 43 | 17.7629 |
| 47 | 30.1334 |
| 44 | 19.4704 |
| 49.5 | 13.9137 |

HRI [μg/week/kg/g/dL]

| PNI | HRI |
| --- | --- |
| 36.5 | 548.363 |
| 42 | 303.03 |
| 37 | 408.263 |
| 40.5 | 559.006 |
| 37 | 300.618 |
| 27 | 913.409 |
| 43.5 | 806.998 |
| 30.5 | 511.108 |
| 33 | 719.281 |
| 36 | 692.042 |
| 35.5 | 389.213 |
| 40 | 242.178 |
| 38 | 347.439 |
| 43.5 | 432.9 |
| 44.5 | 185.469 |
| 40 | 242.895 |
| 36.5 | 234.889 |
| 13.5 | 886.874 |
| 33.5 | 411.184 |
| 40 | 232.504 |
| 40.5 | 501.549 |

**Supplementary Figure S4.** Correlation between CONUT score and body weight of daprodustat (n = 38) (a), roxadustat (n = 21) (b). CONUT, controlling nutritional status; *r_s_*, Spearman's rank correlation coefficient.

**a**

**b**

Daprodustat

Roxadustat

*r_s_* = −0.01

*p* = 0.932

*r_s_* = −0.17

*p* = 0.460

Body weight [kg]

| CONUT score | 体重 |
| --- | --- |
| 7 | 64.8 |
| 3 | 50.35 |
| 5 | 62.25 |
| 3 | 63.5 |
| 4 | 91 |
| 5 | 73.5 |
| 9 | 61.45 |
| 1 | 86.8 |
| 10 | 35.3 |
| 8 | 42.5 |
| 5 | 52.05 |
| 5 | 67.05 |
| 0 | 63.65 |
| 5 | 68.1 |
| 2 | 51.4 |
| 1 | 69.1 |
| 5 | 43.1 |
| 1 | 117.5 |
| 4 | 80.2 |
| 3 | 67.75 |
| 3 | 60.7 |
| 5 | 85.9 |
| 2 | 47.9 |
| 1 | 56 |
| 0 | 74 |
| 6 | 64.9 |
| 3 | 54 |
| 7 | 66.5 |
| 3 | 40.45 |
| 7 | 71.8 |
| 2 | 37 |
| 1 | 53.2 |
| 1 | 57 |
| 7 | 82 |
| 2 | 82.1 |
| 3 | 50.5 |
| 2 | 64.2 |
| 1 | 39 |

Body weight [kg]

| CONUT score | 体重 |
| --- | --- |
| 5 | 65 |
| 1 | 66 |
| 4 | 66.2 |
| 5 | 70 |
| 5 | 59.2 |
| 11 | 35.7 |
| 5 | 47.66 |
| 8 | 63.8 |
| 8 | 55 |
| 8 | 51 |
| 6 | 49.5 |
| 4 | 55.8 |
| 4 | 73.8 |
| 4 | 49 |
| 3 | 55.3 |
| 3 | 53.7 |
| 5 | 51.5 |
| 12 | 59 |
| 6 | 96 |
| 3 | 56.1 |
| 5 | 79.4 |

**Supplementary Table S1.** Correlation coefficient (*r_s_*) between HRI and each factor (baseline)

|  | Daprodustat | | Roxadustat | |
| --- | --- | --- | --- | --- |
|  | *r_s_* | *p* value | *r_s_* | *p* value |
| eGFR | −0.42 | 0.0097 | −0.16 | 0.495 |
| BUN/Cr | −0.06 | 0.718 | −0.06 | 0.802 |
| BMI | −0.06 | 0.700 | 0.10 | 0.665 |
| NLR | 0.44 | 0.006 | 0.44 | 0.047 |
| PLR | 0.32 | 0.051 | 0.36 | 0.115 |
| GNRI | −0.22 | 0.178 | 0.13 | 0.589 |
| CONUT score | 0.47 | 0.006 | 0.37 | 0.104 |
| PNI | −0.42 | 0.009 | −0.27 | 0.232 |
| GPS | 0.25 | 0.162 | 0.27 | 0.243 |
| ALBI score | 0.17 | 0.340 | 0.15 | 0.546 |
| TSAT | 0.25 | 0.180 | 0.16 | 0.609 |
| Ferritin | 0.08 | 0.644 | 0.06 | 0.801 |
| BUN | 0.55 | < 0.001 | 0.29 | 0.200 |
| CRP | 0.18 | 0.312 | 0.10 | 0.657 |
| Albumin | −0.20 | 0.226 | −0.11 | 0.644 |
| TLC | −0.53 | < 0.001 | −0.44 | 0.044 |
| T-cho | −0.05 | 0.804 | −0.17 | 0.454 |
| AST | 0.05 | 0.756 | 0.10 | 0.657 |
| ALT | 0.22 | 0.184 | 0.06 | 0.803 |
| I-Bil | −0.08 | 0.726 | −0.25 | 0.386 |
| T-Bil | −0.22 | 0.214 | −0.01 | 0.983 |
| MCV | −0.10 | 0.535 | −0.39 | 0.081 |

Data are shown as Spearman's rank correlation coefficient. It was excluded from the analysis due to missing data, when HIF-PHIs were started at another hospital or clinic and no medical information was available. HRI, HIF-PH inhibitor resistance index; eGFR, estimated glomerular filtration rate; BUN/Cr, blood urea nitrogen to creatinine ratio; BMI, body mass index; NLR, neutrophil to lymphocyte ratio; PLR, platelet to lymphocyte ratio; GNRI, geriatric nutritional risk index; CONUT, controlling nutritional status; PNI, prognostic nutritional index; GPS, glasgow prognostic score; ALBI, albumin-bilirubin; TSAT, transferrin saturation; CRP, C-reactive protein; TLC, total lymphocyte count; T-cho, total cholesterol; AST, aspartate aminotransferase; ALT, alanine aminotransferase; I-Bil, indirect bilirubin; T-Bil, total bilirubin; MCV, mean corpuscular volume *r_s_*, Spearman's rank correlation coefficient.

**Supplementary Table S2.** Patient characteristics used for HRI calculation

|  | Daprodustat (n = 38) | Roxadustat (n = 21) |
| --- | --- | --- |
| Hb [g/dL] | 10.4 (9.3–11.0) | 11.1 (9.2–11.5) |
| Ferritin [ng/mL] | 146 (80–206) | 172 (68–248) |
| TSAT [%] | 24.2 (18.5–33.2) | 21.6 (14.3–25.4) |
| HIF-PHI dose [mg] | 4 (2–6) | 100 (40–150) |
| Evaluated period [day] | 61 (41–124) | 33 (28–53) |
| HRI [μg/week/kg/Hb] | 37.3 (23.0–67.5) | 411.2 (300.6–559.0) |

Data are shown as median (IQR). The evaluated period refers to the time from the start of the evaluation dose to the time of evaluation. IQR, interquartile range; HRI, HIF-PH inhibitor resistance index; Hb, hemoglobin; TSAT, transferrin saturation.

**Supplementary Table S3.** Factors associated with the above-median HRI of daprodustat

|  | Univariate analysis | | |  | Multivariate analysis | | |
| --- | --- | --- | --- | --- | --- | --- | --- |
|  | OR | 95% CI | *p* value |  | OR | 95% CI | *p* value |
| Age ≥ 75 years | 1.00 | 0.28–3.54 | 1.000 |  |  |  |  |
| Female | 0.40 | 0.10–1.55 | 0.183 |  |  |  |  |
| Baseline eGFR [mL/min/1.73 m^2^] | 0.86 | 0.76–0.97 | 0.012 |  | 0.92 | 0.82–1.04 | 0.180 |
| Baseline Ferritin [ng/mL] | 1.00 | 0.99–1.00 | 0.429 |  |  |  |  |
| Baseline TSAT [%] | 1.03 | 0.96–1.11 | 0.443 |  |  |  |  |
| Diabetic comorbidity | 0.53 | 0.14–1.92 | 0.330 |  |  |  |  |
| CKD etiology: Diabetes | 0.32 | 0.07–1.51 | 0.150 |  |  |  |  |
| CKD etiology: Hypertension | 0.77 | 0.19–3.16 | 0.721 |  |  |  |  |
| CKD etiology: AD/GN/Vasculitis | 3.92 | 0.68–22.7 | 0.127 |  |  |  |  |
| Peritoneal dialysis | 3.11 | 0.66–14.6 | 0.150 |  |  |  |  |
| Post-cure malignancy | 0.70 | 0.13–3.68 | 0.676 |  |  |  |  |
| History of ESA use | 3.71 | 0.97–14.2 | 0.056 |  |  |  |  |
| Abnormal MCV | 2.27 | 0.36–14.2 | 0.382 |  |  |  |  |
| Use of antacids | 1.54 | 0.42–5.64 | 0.512 |  |  |  |  |
| Iron therapy | 1.86 | 0.52–6.85 | 0.332 |  |  |  |  |
| Drug interactions | 0.70 | 0.13–3.68 | 0.676 |  |  |  |  |
| CONUT score [per-1 increase] | 2.53 | 1.44–4.47 | 0.001 |  | 2.39 | 1.31–4.35 | 0.005 |

Adjusted odds ratios for the above-median HRI of daprodustat (n = 38) were estimated using logistic regression analysis. Selected covariates were those statistically significant in univariate analysis using the forced entry method. VIF < 5.0, confirming no multicollinearity of the explanatory variables. Abnormal MCV was defined as MCV <80 fL or MCV >100 fL. Antacids included proton pump inhibitors, potassium-competitive acid blockers, and histamine H2 receptor antagonists. Drug interactions were due to CYP2C8 inhibition. OR, odds ratio; CI, confidence interval; HRI, HIF-PH inhibitor resistance index; eGFR, estimated glomerular filtration rate; CKD, chronic kidney disease; AD, autoimmune disease; GN, glomerulonephritis; ESA, erythropoiesis-stimulating agent; MCV, mean corpuscular volume; CONUT, controlling nutritional status; VIF, variance inflation factor; CYP, cytochrome P450.

**Supplementary Table S4.** Factors associated with the log-transformed HRI of daprodustat

|  | Univariate analysis | | | |  | Multivariate analysis | | | |
| --- | --- | --- | --- | --- | --- | --- | --- | --- | --- |
|  | Β | SE | *t* | *p* value |  | Β | SE | *t* | *p* value |
| Age ≥ 75 years | −0.14 | 0.26 | −0.53 | 0.598 |  |  |  |  |  |
| Female | 0.03 | 0.27 | 0.10 | 0.921 |  |  |  |  |  |
| Baseline eGFR [mL/min/1.73 m^2^] | −0.04 | 0.01 | −2.59 | 0.014 |  | −0.00 | 0.01 | −0.15 | 0.884 |
| Baseline Ferritin [ng/mL] | −0.00 | 0.00 | −0.17 | 0.867 |  |  |  |  |  |
| Baseline TSAT [%] | 0.02 | 0.01 | 1.74 | 0.093 |  |  |  |  |  |
| Diabetic comorbidity | −0.11 | 0.26 | −0.42 | 0.680 |  |  |  |  |  |
| CKD etiology: Diabetes | −0.53 | 0.28 | −1.91 | 0.063 |  |  |  |  |  |
| CKD etiology: Hypertension | −0.29 | 0.28 | −1.02 | 0.312 |  |  |  |  |  |
| CKD etiology: AD/GN/Vasculitis | 0.73 | 0.29 | 2.48 | 0.018 |  | −0.08 | 0.21 | −0.38 | 0.703 |
| Peritoneal dialysis | 0.26 | 0.29 | 0.90 | 0.376 |  |  |  |  |  |
| Post-cure malignancy | 0.04 | 0.33 | 0.12 | 0.906 |  |  |  |  |  |
| History of ESA use | 0.60 | 0.24 | 2.50 | 0.017 |  | 0.27 | 0.16 | 1.62 | 0.114 |
| Abnormal MCV | 0.51 | 0.34 | 1.50 | 0.143 |  |  |  |  |  |
| Use of antacids | 0.29 | 0.26 | 1.11 | 0.274 |  |  |  |  |  |
| Iron therapy | 0.30 | 0.25 | 1.18 | 0.248 |  |  |  |  |  |
| Drug interactions | 0.15 | 0.33 | 0.47 | 0.644 |  |  |  |  |  |
| CONUT score [per-1 increase] | 0.25 | 0.03 | 8.45 | < 0.001 |  | 0.24 | 0.04 | 6.59 | < 0.001 |

Regression coefficients for ln(HRI) of daprodustat (n = 38) were estimated using linear regression analysis. Selected covariates were those statistically significant in univariate analysis using the forced entry method. The adjusted R² of the model was 0.66. Normality of residuals was confirmed by the Shapiro–Wilk test (*p* = 0.09). VIF < 5.0, confirming no multicollinearity of the explanatory variables. Abnormal MCV was defined as MCV <80 fL or MCV >100 fL. Antacids included proton pump inhibitors, potassium-competitive acid blockers, and histamine H2 receptor antagonists. Drug interactions were due to CYP2C8 inhibition. SE, standard error; HRI, HIF-PH inhibitor resistance index; eGFR, estimated glomerular filtration rate; CKD, chronic kidney disease; AD, autoimmune disease; GN, glomerulonephritis; ESA, erythropoiesis-stimulating agent; MCV, mean corpuscular volume; CONUT, controlling nutritional status; VIF, variance inflation factor; CYP, cytochrome P450.

**Supplementary Table S5.** Factors associated with the above-median HRI of roxadustat

|  | Univariate analysis | | |
| --- | --- | --- | --- |
|  | OR | 95% CI | *p* value |
| Age ≥ 75 years | 1.14 | 0.17–7.60 | 0.890 |
| Female | 1.93 | 0.25–14.9 | 0.529 |
| Baseline eGFR [mL/min/1.73 m^2^] | 0.98 | 0.92–1.06 | 0.646 |
| Baseline Ferritin [ng/mL] | 1.00 | 1.00–1.01 | 0.441 |
| Baseline TSAT [%] | 1.02 | 0.95–1.09 | 0.670 |
| Diabetic comorbidity | 3.00 | 0.41–21.9 | 0.279 |
| CKD etiology: Diabetes | 1.12 | 0.13–9.94 | 0.916 |
| CKD etiology: Hypertension | 1.93 | 0.25–14.9 | 0.529 |
| CKD etiology: AD/GN/Vasculitis | 3.00 | 0.41–21.9 | 0.279 |
| Peritoneal dialysis | 0.36 | 0.06–2.16 | 0.262 |
| Post-cure malignancy | 4.50 | 0.63–32.3 | 0.135 |
| History of ESA use | 0.88 | 0.13–5.82 | 0.890 |
| Abnormal MCV | 0.44 | 0.06–3.16 | 0.413 |
| Use of antacids | 2.29 | 0.32–16.5 | 0.413 |
| Iron therapy | 0.56 | 0.10–3.15 | 0.507 |
| CONUT score [per-1 increase] | 2.55 | 1.03–6.36 | 0.044 |

Odds ratios for the above-median HRI of roxadustat (n = 21) were estimated using logistic regression analysis. Abnormal MCV was defined as MCV <80 fL or MCV >100 fL. Antacids included proton pump inhibitors, potassium-competitive acid blockers, and histamine H2 receptor antagonists. OR, odds ratio; CI, confidence interval; HRI, HIF-PH inhibitor resistance index; eGFR, estimated glomerular filtration rate; CKD, chronic kidney disease; AD, autoimmune disease; GN, glomerulonephritis; ESA, erythropoiesis-stimulating agent; MCV, mean corpuscular volume; CONUT, controlling nutritional status.

**Supplementary Table S6.** Factors associated with the log-transformed HRI of roxadustat

|  | Univariate analysis | | | |  | Multivariate analysis | | | |
| --- | --- | --- | --- | --- | --- | --- | --- | --- | --- |
|  | Β | SE | *t* | *p* value |  | Β | SE | *t* | *p* value |
| Age ≥ 75 years | −0.03 | 0.24 | −0.12 | 0.906 |  |  |  |  |  |
| Female | −0.05 | 0.25 | −0.21 | 0.833 |  |  |  |  |  |
| Baseline eGFR [mL/min/1.73 m^2^] | −0.01 | 0.01 | −1.22 | 0.237 |  |  |  |  |  |
| Baseline Ferritin [ng/mL] | 0.00 | 0.00 | 1.25 | 0.229 |  |  |  |  |  |
| Baseline TSAT [%] | 0.01 | 0.01 | 0.75 | 0.469 |  |  |  |  |  |
| Diabetic comorbidity | 0.24 | 0.23 | 1.03 | 0.318 |  |  |  |  |  |
| CKD etiology: Diabetes | 0.03 | 0.27 | 0.11 | 0.912 |  |  |  |  |  |
| CKD etiology: Hypertension | 0.10 | 0.25 | 0.39 | 0.699 |  |  |  |  |  |
| CKD etiology: AD/GN/Vasculitis | 0.33 | 0.22 | 1.45 | 0.164 |  |  |  |  |  |
| Peritoneal dialysis | −0.15 | 0.21 | −0.73 | 0.476 |  |  |  |  |  |
| Post-cure malignancy | 0.49 | 0.20 | 2.46 | 0.023 |  | 0.15 | 0.17 | 0.89 | 0.385 |
| History of ESA use | 0.29 | 0.23 | 1.25 | 0.226 |  |  |  |  |  |
| Abnormal MCV | −0.04 | 0.24 | −0.18 | 0.860 |  |  |  |  |  |
| Use of antacids | 0.25 | 0.23 | 1.07 | 0.298 |  |  |  |  |  |
| Iron therapy | −0.25 | 0.21 | −1.19 | 0.250 |  |  |  |  |  |
| Drug interactions | 0.18 | 0.50 | 0.36 | 0.722 |  |  |  |  |  |
| CONUT score [per-1 increase] | 0.14 | 0.03 | 5.16 | < 0.001 |  | 0.12 | 0.03 | 4.02 | < 0.001 |

Regression coefficients for ln(HRI) of roxadustat (n = 21) were estimated using linear regression analysis. Selected covariates were those statistically significant in univariate analysis using the forced entry method. The adjusted R² of the model was 0.56. Normality of residuals was confirmed by the Shapiro–Wilk test (*p* = 0.77). VIF < 5.0, confirming no multicollinearity of the explanatory variables. Abnormal MCV was defined as MCV <80 fL or MCV >100 fL. Antacids included proton pump inhibitors, potassium-competitive acid blockers, and histamine H2 receptor antagonists. Drug interactions were due to CYP2C8 inhibition. SE, standard error; HRI, HIF-PH inhibitor resistance index; eGFR, estimated glomerular filtration rate; CKD, chronic kidney disease; AD, autoimmune disease; GN, glomerulonephritis; ESA, erythropoiesis-stimulating agent; MCV, mean corpuscular volume; CONUT, controlling nutritional status; VIF, variance inflation factor; CYP, cytochrome P450.
